# The carboxyl terminal mutational hotspot of the ciliary disease protein RPGR^ORF15^ (retinitis pigmentosa GTPase regulator) is glutamylated *in vivo*

**DOI:** 10.1242/bio.016816

**Published:** 2016-03-03

**Authors:** Kollu N. Rao, Manisha Anand, Hemant Khanna

**Affiliations:** Department of Ophthalmology, Horae Gene Therapy Center, UMASS Medical School, Worcester, MA 01605, USA

**Keywords:** Retina, RPGR, Cilia, Glutamylation, GT335

## Abstract

Mutations in *RPGR^ORF15^* (retinitis pigmentosa GTPase regulator) are a major cause of inherited retinal degenerative diseases. RPGR^ORF15^ (1152 residues) is a ciliary protein involved in regulating the composition and function of photoreceptor cilia. The mutational hotspot in RPGR^ORF15^ is an unusual C-terminal domain encoded by exon ORF15, which is rich in polyglutamates and glycine residues (Glu-Gly domain) followed by a short stretch of basic amino acid residues (RPGR^C2^ domain; residues 1072-1152). However, the properties of the ORF15-encoded domain and its involvement in the pathogenesis of the disease are unclear. Here we show that RPGR^ORF15^ is glutamylated at the C-terminus, as determined by binding to GT335, which recognizes glutamylated substrates. This reactivity is lost in two mouse mutants of *Rpgr*, which do not express RPGR^ORF15^ due to disease-causing mutations in exon ORF15. Our results indicate that RPGR^ORF15^ is posttranslationally glutamylated in the Glu-Gly domain and that the GT335 antibody predominantly recognizes RPGR^ORF15^ in photoreceptor cilia.

## Abstract (precis)

**Summary:** This study shows that the mutational hotspot of ciliary protein RPGR^ORF15^, commonly associated with severe blindness, is posttranslationally glutamylated at its C-terminus and is a target of GT335.

## INTRODUCTION

Retinitis pigmentosa (RP) is a group of genetically and clinically heterogeneous disorders of the eye. RP is characterized by night blindness due to the loss of rod photoreceptors, followed by complete blindness due the loss of cones (Bird, 1987; Fishman, 1978). RP is inherited in autosomal dominant, autosomal recessive, as well as X-linked manner, with over 200 causative genes identified to date (https://sph.uth.edu/retnet/). X-linked RP is one of the most severe forms with symptoms starting as early as in the first decade of life, which progress into complete blindness usually by the second decade of life (Fishman et al., 1988; Heckenlively et al., 1988). Mutations in two genes, *RPGR* and *RP2*, account for >80% of XLRP cases. Of these, *RPGR* mutations are found in ∼70% of cases. Moreover, 15-20% of simplex RP patients carry mutations in *RPGR*. These data make RPGR a common cause of RP, accounting for ∼20% of all RP cases (Churchill et al., 2013; Daiger et al., 2007).

Photoreceptors are polarized neurons with a distinct inner segment (IS) involved in protein synthesis and trafficking and photosensory outer segment (which is loaded with proteins involved in phototransduction). The outer segment extends from the apical region of the inner segment in the form of a narrow bridge-like structure called microtubule-based sensory or connecting cilium (Besharse and Bok, 2011). RPGR^ORF15^ localizes to the connecting cilium and is likely involved in regulating the composition of the outer segment (Anand and Khanna, 2012; Rao et al., 2015).

RPGR is extensively alternatively spliced; however, there are two major RPGR isoforms: constitutive RPGR (RPGR^const^) and RPGR^ORF15^. Whereas the RPGR^const^ isoform encodes exons 1-19 of the *RPGR* gene (amino acids 1-815), the RPGR^ORF15^ isoform terminates in an alternate exon ORF15, which includes exon 15 and part of intron 15 of RPGR (amino acids 1-1152) (Anand and Khanna, 2012). Both variants share a common N-terminal domain (encoded by exons 1-15) (Murga-Zamalloa et al., 2010). On the other hand, the C-terminal domain of RPGR^const^ encoded by exons 16-19 carries an isoprenylation motif (residues 812-815) whereas RPGR^ORF15^ terminates in a long intron 15, which is a purine-rich region encoding a glutamic acid-glycine (Glu-Gly)-rich acidic domain (Vervoort et al., 2000). This domain is followed by a short stretch of basic amino acids, termed RPGR^C2^ domain (residues 1071-1152). Mutation analysis revealed that exon ORF15 is a mutational hotspot, accounting for 50-60% of XLRP cases (Vervoort et al., 2000). The majority of human disease-causing mutations in this exon are frameshift or nonsense variations, which result in a premature stop codon, whereas in-frame deletions or duplications or missense changes are tolerated.

Mouse and canine models of *Rpgr* have also been reported. An *Rpgr^null^* mouse was generated by interrupting exons 4-6 of the *Rpgr* gene and was predicted to affect the expression of all RPGR isoforms (Hong et al., 2000). More recently, a naturally occurring *Rpgr^rd9^* mouse model was characterized; this mouse carries a frameshift mutation in exon ORF15 resulting in a premature stop but does not seem to affect the expression of the RPGR^const^ isoform (Thompson et al., 2012). Two canine models carrying mutations in exon ORF15 have also been reported (Zhang et al., 2002). These models represent considerable phenotypic variability, which is consistent with heterogenic clinical presentation of *RPGR^ORF15^* patients.

Being a mutational hotspot, it is important to evaluate the properties of exon ORF15 of *RPGR*. In this study, we hypothesized that the polyglutamate rich domain of RPGR^ORF15^ exhibits similar properties as the glutamate-rich regions of α-tubulin, whose C-terminal glutamate residues are posttranslationally glutamylated specifically in cilia.

## RESULTS

### Posttranslational modification of tubulin in the absence of RPGR^ORF15^

**Fig. 1.**
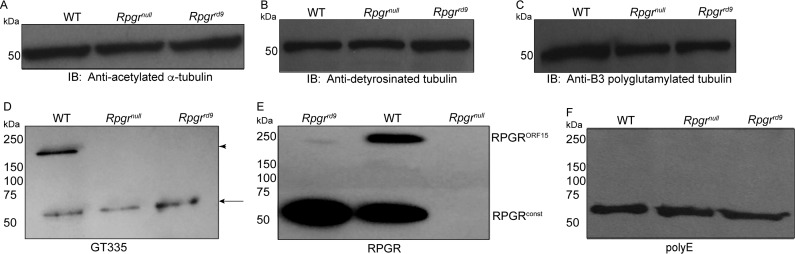
**GT335 detects RPGR^ORF15^.** (A-C) Immunoblot (IB) analysis of retinal extracts from wild type (WT), *Rpgr^null^* and *Rpgr^rd9^* mice was performed using antibodies against indicated forms of tubulin. An equal amount of protein extract (30 µg) was loaded in each lane. (D-F) Retinal extracts from indicated mouse strains were analyzed by SDS-PAGE and immunoblotting using anti-GT335 (D), anti-RPGR (E) or anti-polyE (F) antibodies. Arrow in D indicates the tubulin-reactive band whereas arrowhead points to the RPGR^ORF15^ band. Three independent replicates were performed for these experiments.

Microtubules are polymers of α/β tubulin heterodimers (Mitchison and Kirschner, 1984). Tubulins undergo diverse posttranslational modifications in an organelle or cellular substructure-specific manner (Verhey and Gaertig, 2007). For example, ciliary microtubules are enriched in acetylation, detyrosination and glutamylation (Yu et al., 2015). These modifications regulate the structure and function of the microtubule cytoskeleton (Mendes Maia et al., 2014; O'hagan and Barr, 2012). Given that RPGR associates with microtubule-based assemblies and that loss of RPGR alters microtubule-based photoreceptor ciliary trafficking (Anand and Khanna, 2012; Rao et al., 2015), we examined whether tubulin modifications are altered in the absence of RPGR. To this end, we used two *Rpgr*-mutant lines; *Rpgr^null^* and *Rpgr^rd9^*. The *Rpgr^null^* mouse does not exhibit expression of RPGR^const^ and RPGR^ORF15^, whereas the *Rpgr^rd9^* mice only express the RPGR^const^ (∼90 kDa) isoform (Rao et al., 2015; Thompson et al., 2012). Immunoblot analysis of retinal extracts from wild-type (WT), *Rpgr^null^*, and *Rpgr^rd9^* mouse retinas using antibodies against various post-translationally modified tubulin revealed no changes in the levels of acetylated α-tubulin, detyrosinated tubulin or glutamylated tubulin (B3 and GT335) (Fig. 1A-D).

### RPGR^ORF15^ is a target of GT335

During our analysis, we found that the GT335 antibody, in addition to detecting the glutamylated tubulin-specific band at ∼50 kDa, recognized a higher molecular weight band (∼200 kDa) in WT mouse retinal extracts (Fig. 1D). This band was of the same molecular weight as the RPGR^ORF15^-immunoreactive band, as determined by western blotting using anti-RPGR antibody (Fig. 1E). We did not detect a similar immunoreactive band (∼200 kDa) using B3 antibody (not shown). Previous studies showed that in addition to tubulins, GT335 recognizes other targets of glutamylation, such as nucleosome assembly proteins, NAP1 and NAP2 (Regnard et al., 2000). However, B3 antibody specifically detects polyglutamylated α-tubulin (Van Dijk et al., 2007).

The GT335 antibody was raised against an octapeptide EGEGE*EEG, which is modified by the addition of two glutamyl subunits on the fifth E (*). The C-terminus of RPGR^ORF15^, on the other hand, predominantly carries GEEEEG and GEEEG repeats. These repeats could potentially be substrates for glutamylation. We thus asked whether the GT335 antibody cross-reacts with an unknown protein of the same molecular weight as RPGR^ORF15^ or specifically recognizes the C-terminal domain of RPGR^ORF15^. We examined the expression of this band in the *Rpgr^null^* and the *Rpgr^rd9^* retinas. Our hypothesis was that if this band were a cross-reacting species, then we would observe it even in the absence of RPGR^ORF15^; however, if this reactivity were specific, then we would not observe this high molecular weight band in *Rpgr^rd9^* (frameshift mutation in exon ORF15) and *Rpgr^null^* retinal extracts. Immunoblot analysis revealed that the GT335-immunoreactive band was undetectable in the *Rpgr^null^* and *Rpgr^rd9^* retinal extracts (Fig. 1D). The GT335 antibody is a well-characterized antibody, which specifically recognizes the first branch point glutamate added to the target residue (Van Dijk et al., 2007; Wolff et al., 1992). Thus, this reactivity does not distinguish between mono- and polyglutamylated RPGR^ORF15^. To clarify this, we performed immunoblot analysis using polyE antibody, which recognizes long polyglutamylated side chains (Magiera and Janke, 2013). As shown in Fig. 1F, polyE antibody did not detect the high molecular band; however, tubulin-specific bands at ∼50 kDa were detected. These results indicate that RPGR^ORF15^ is most likely monoglutamylated.

### RPGR^ORF15^ associates with GT335 in the retina

**Fig. 2.**
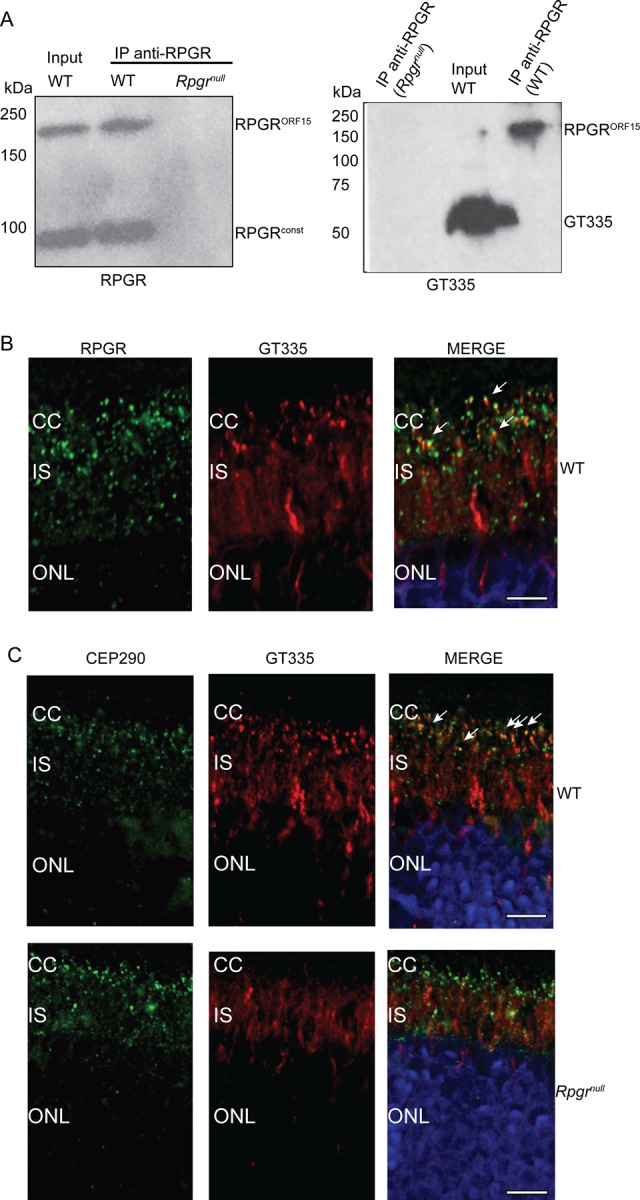
**GT335 association and reactivity in *Rpgr^null^* retina.** (A) Retinal extracts from wild type (WT) and *Rpgr^null^* mice were subjected to immunoprecipitation (IP) using anti-RPGR antibody followed by SDS-PAGE and immunoblotting using anti-RPGR (left) or GT335 (right) antibody. Input lane shows 10% of the protein extract used in IP. (B,C) Cryosections of wild type (WT; B; upper panel, C) and 2-month-old *Rpgr^null^* (C) mouse retinas were stained with anti-RPGR (B; green) or anti-CEP290 (panel C; green), and anti-GT335 (red; B,C) antibodies. Arrows in merge point to co-localization of RPGR or CEP290 with GT335-specific signal. Scale bar: 2 µm in B; 5 µm in C. Nuclei were stained with Hoechst (blue). CC, connecting cilium; IS, photoreceptor inner segment; ONL, photoreceptor outer nuclear layer. These experiments were repeated three times with independent mouse retinal samples.

To further validate the binding of GT335 to RPGR^ORF15^, we performed immunoprecipitation from mouse retina using anti-RPGR^ORF15^ antibody followed by immunoblotting using GT335 antibody or anti-RPGR^ORF15^ antibody. RPGR immunoprecipitation pulled down both RPGR^ORF15^ and RPGR^const^ isoforms from the wild type retinal extracts but not from *Rpgr^null^* mice; however, the GT335-immunoreactive band coincided with anti-RPGR^ORF15^-reactive band (Fig. 2A, right panel). Furthermore, immunofluorescence analysis of wild-type mouse retina using anti-RPGR and GT335 antibodies showed that they co-localize at the connecting cilium (CC) (Fig. 2B).

### Immunofluorescence analysis of GT335 in *Rpgr^null^* mouse retina

GT335 recognizes polyglutamylated tubulin in microtubules, which are abundant in the cilia. To test whether the ciliary staining of GT335 is altered in the absence of RPGR, we performed immunofluorescence analysis of wild-type and *Rpgr^null^* mouse retina. Our analysis revealed reduced staining of GT335 in the *Rpgr^null^* photoreceptor connecting cilium. As shown in Fig. 2C, co-localization of GT335 with anti-CEP290 (a connecting cilium marker; arrows pointing to yellow spots) (Murga-Zamalloa et al., 2011) is observed in wild type retinal sections but not in the *Rpgr^null^* retinas, which predominantly exhibits green signal corresponding to anti-CEP290 antibody, in the connecting cilium. These data indicate that in photoreceptor cilia, the ciliary staining of GT335 is largely mediated by binding to glutamylated RPGR. Such staining is diminished in the *Rpgr^null^* mouse retina.

### Human RPGR^ORF15^ is immunoreactive to GT335

**Fig. 3.**
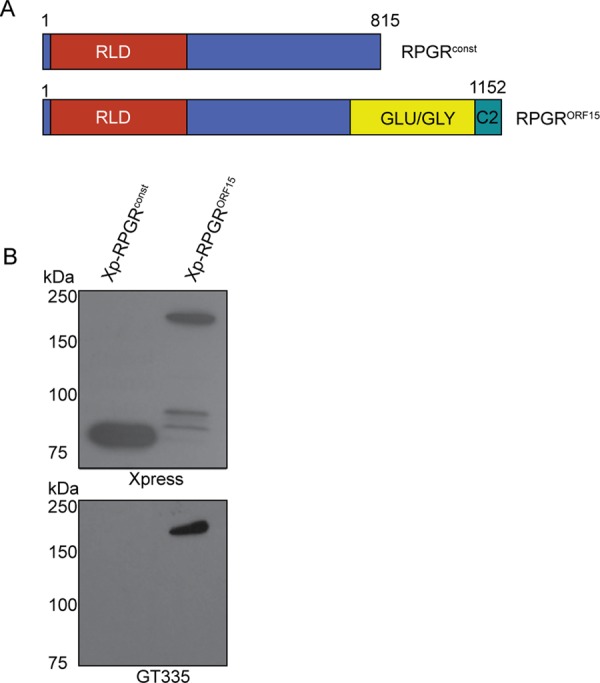
**Human RPGR^ORF15^ reacts with GT335.** (A) This panel depicts a schematic representation of the human RPGR^const^ and RPGR^ORF15^ isoforms. Numbers denote the positons of amino acid residues. RLD, RCC1-like domain; Glu-Gly, glutamic acid and glycine rich domain. (B) hTERT-RPE1 cells were transiently transfected with constructs encoding Xp-RPGR^const^ or Xp-RPGR^ORF15^. An equal amount of cell extract was analyzed by SDS-PAGE and immunoblotting using anti-Xpress or GT335 antibody. All experiments were repeated three independent times.

The studies described above were carried out using mouse retinal extracts. We also wanted to validate these findings in human RPGR protein. Using immunoreactivity to GT335 as a tractable assay system, we asked whether GT335 also recognizes both major variants of human RPGR: RPGR^const^ and RPGR^ORF15^ protein. We transiently transfected hTERT-RPE1 cells with mammalian expression constructs encoding Xpress (Xp)-tagged human RPGR^const^ and RPGR^ORF15^ (Fig. 3A), followed by SDS-PAGE and immunoblotting of protein extracts using anti-Xp or GT335 antibodies. As shown in Fig. 3B, GT335-immunoreactivity was detected only in cells expressing Xp-RPGR^ORF15^. Immunoblotting using anti-Xpress antibody validated the expression of the recombinant human RPGR proteins. These results suggest that the C-terminal glutamic acid rich domain of RPGR^ORF15^ is a likely target for glutamylation.

### Disease-causing mutations in RPGR^ORF15^ alter its binding to GT335

**Fig. 4.**
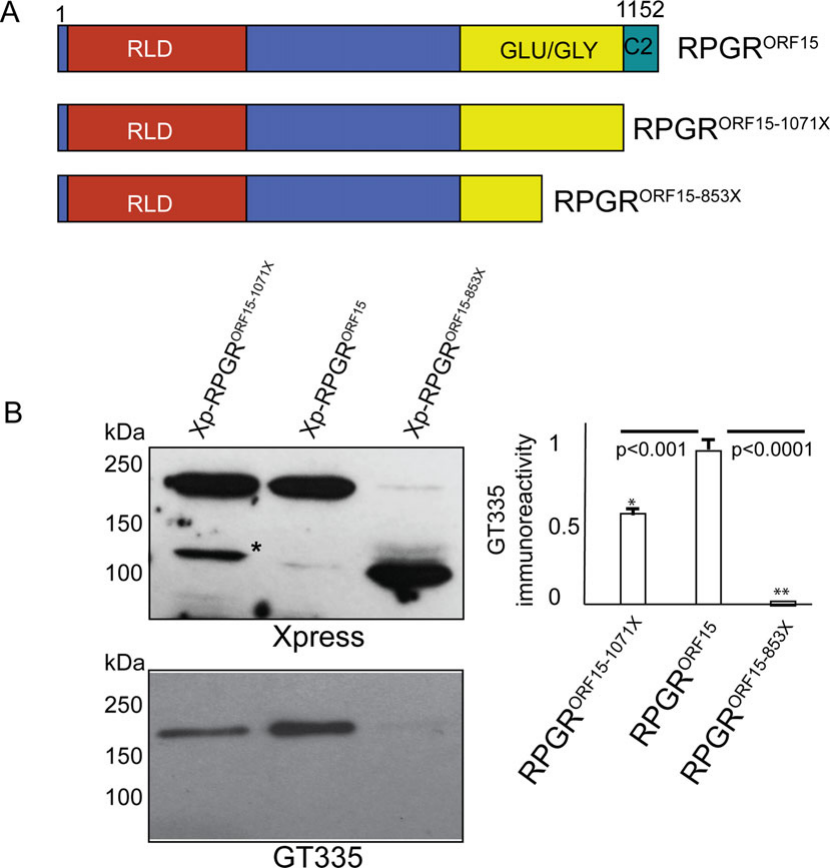
**Human disease mutations in RPGRORF15 alter binding to GT335.** (A) Schematic representation of the location of the human exon ORF15 mutations. RLD, RCC1-like domain; Glu-Gly, glutamic acid and glycine rich domain; C2, RPGR^C2^ domain. (B) hTERT-RPE1 cells were transiently transfected with constructs encoding Xpress-tagged full-length RPGR^ORF15^ or two disease-causing mutants; RPGR^ORF15-1071X^ or RPGR^ORF15-853X^. Equal amount of protein extracts from these cells were analyzed by SDS-PAGE and immunoblotting using anti-Xpress (upper panel) or anti-GT335 (lower panel) antibody. Asterisk indicates degraded protein product. Densitometric analysis was performed to quantify the immunoblot signal. The data are represented as ratio relative to the intensity of GT335-immunoreactivity with full-length RPGR^ORF15^, set as 1. Error bars represent standard deviation. *P<0.001; **P<0.0001. Data are representative of three independent experiments.

We hypothesized that disease-associated mutations in exon ORF15, which alter the polyglutamate repeats affect its glutamylation and thus, reactivity to GT335. To test this, we transiently transfected hTER-RPE1 cells with constructs encoding Xp-tagged RPGR^ORF15^ and truncated versions of RPGR^ORF15^: RPGR^ORF15^-1071X and RPGR^ORF15^-853X (Fig. 4A). Immunoblot analysis of protein extracts using GT335 antibody revealed that whereas full length RPGR^ORF15^ was recognized by GT335, RPGR^ORF15^-Glu1071X exhibited ∼50% reactivity and RPGR^ORF15^-Glu853X exhibited only ∼0.1% immunoreactivity to GT335 (Fig. 4B).

## DISCUSSION

Our studies reveal two key findings: (i) a novel molecule that is recognized by GT335. In fact, RPGR^ORF15^ is likely the fifth molecule that is recognized by GT335; the first four being α-tubulin, β-tubulin, NAP1 and NAP2 (Regnard et al., 2000); (ii) sequence alterations in the Glu-Gly domain of RPGR^ORF15^ alter its glutamylation. However, the function of glutamylated RPGR^ORF15^ is not known. We propose that glutamylated RPGR^ORF15^ regulates the integrity of the multiprotein complexes at the cilium and modulates their entry or retention inside the cilium. However, human disease mutations in the polyglutamate-rich region appear to alter the length of this region (and the amount of polyglutamates) and are associated with relatively mild retinal dysfunction in patients. Thus, alterations in the glutamylation of RPGR^ORF15^ may compromise but not eliminate its function. Evaluation of complete loss of the ORF15 domain on ciliary function will provide insights into the precise role of glutamylation of RPGR^ORF15^.

What is the mechanism of glutamylation of RPGR^ORF15^? Glutamylation is an evolutionarily conserved and is a widely distributed modification involved in diverse functions (Janke et al., 2008; Van Dijk et al., 2008). Moreover, tubulin glutamylation occurs at the C-terminal glutamate-rich region (Edde et al., 1990) and is carried out by tubulin tyrosine ligase-like (TTLL) family of proteins (Raunser and Gatsogiannis, 2015). Recently, a member of the TTLL family, TTLL5, was reported to be involved in human retinal degeneration (Sergouniotis et al., 2014). We propose that TTLL5 or another TTLL family member is a candidate glutamylase for RPGR^ORF15^. A *Ttll5^ko^* mouse model was reported to have sperm defects; however, retinal defects were not detected in this model (Lee et al., 2013). It is possible that the defects could occur at ages older than those tested in that study. Further studies to test these scenarios have the potential to reveal critical insights into the pathogenesis of retinal degeneration due to defective posttranslational modifications of ciliary proteins. Our results showing that RPGR^ORF15^-Glu1071X exhibited ∼50% reactivity and RPGR^ORF15^-Glu853X exhibited only ∼0.1% immunoreactivity to GT335 suggest the involvement of C2 domain in efficient glutamylation of RPGR^ORF15^; however, both the Glu-Gly domain and the C2 domain may be critical for glutamylation of RPGR^ORF15^. We propose that the C2 domain may act as a binding site for the enzymes and that both the Glu-Gly domain and C2 domain work in concert for efficient binding and glutamylation reactions.

Although our data indicate that RPGR^ORF15^ is likely monoglutamylated, we cannot rule out the possibility that RPGR^ORF15^ is polyglutamylated. This is because the C-terminus of RPGR^ORF15^ predominantly carries GEEEEG and GEEEG repeats, which could be glutamylated in tandem. However, identification of the glutamylase will provide further insights into the glutamylation status of RPGR^ORF15^. For example, TTLL4, TTLL5, and TTLL7 are side-chain initiating polyglutamylases, TTLL6, TTLL11 and TTLL13 preferentially elongate the side chains. Nonetheless, glutamylation of RPGR^ORF15^ may alter the function of RPGR by modulating its ability to interact with other proteins at the cilium due to changes in the net charge of the Glu-Gly domain. Further studies are underway to assess the effect of glutamylation of RPGR^ORF15^ on its ability to maintain the integrity of its interactome and/or interact with additional proteins. Overall, these studies will provide new insights into the mode of regulation of ciliary protein trafficking and pathogenesis of associated ciliopathies.

## MATERIALS AND METHODS

### Animals

All studies involving animals were approved by the Institutional Animal Care and Use Committee of UMASS Medical School. Wild type C57BL6/J and *Rpgr^rd9^* mice were procured from Jackson Labs; *Rpgr^null^* mice were obtained from Dr Tiansen Li (National Eye Institute). Only hemizygous male mice were used in this study as *Rpgr* is an X-linked gene.

### Antibodies

The GT335 (1:500 dilution) and polyE (1:200 dilution) antibodies were obtained from AdipoGen (San Diego, CA); anti-anti-acetylated α-tubulin (1:1000 dilution) and anti-polyglutamylated tubulin B3 (1:500 dilution) were obtained from Sigma-Aldrich; anti-detyrosinated antibody was obtained from Abcam; anti-Xpress antibody (1:200 dilution) was procured from Invitrogen. Anti-RPGR antibody (1:500 dilution) was raised against an N-terminal epitope, which is common to both RPGR^const^ and RPGR^ORF15^ isoforms. Detailed characterization of this antibody was previously reported (Ghosh et al., 2010; Rao et al., 2015).

### Plasmids, cell culture, transient transfection and IP

The human cDNA encoding full-length human RPGR^const^ and RPGR^ORF15^ were cloned into pcDNA4 (Invitrogen), which expressed N-terminally X-press-tagged recombinant proteins, and sequence verified. hTERT-RPE1 cells (ATCC) were maintained in DMEM/F12 (Invitrogen) supplemented with 10% fetal bovine serum and penicillin/streptomycin and tested for contamination. Transient transfections were performed using Lipofectamine 2000 (Invitrogen). Cells were lysed 48 h post-transfection in lysis buffer containing 25 mM Tris, 150 mM NaCl, 1 mM EDTA, 1% NP-40, 5% glycerol (pH 7.4) with Complete protease inhibitors (Roche) and lysates were spun at 18,000×***g*** for 15 min at 4°C. Equal amount of protein was analyzed by SDS-PAGE and immunoblotting, as described (Khanna et al., 2005). Immunoprecipitation of mouse retinal extracts was performed, as described (Khanna et al., 2005). All experiments were repeated three independent times.

### Immunofluorescence

Staining of mouse retina was performed as described (Li et al., 2013). Cryosections of mouse retinas fixed in 4% paraformaldehyde were permeabilized and blocked using 5% normal goat serum followed by incubation with primary antibody overnight at 4°C. Slides were then washed three times with phosphate buffered saline and further incubated with secondary antibody for 1 h at room temperature. Hoechst 33342 (Life Technologies) was added (1 µg/ml) to label the nuclei and the sections were then mounted (Fluoromount; Electron Microscopy Services, Hatfield, PA) under glass coverslips and visualized using Leica TCS Sp5 II laser microscope (Leica Microsystems).
